# Gingivectomy–Gingivoplasty for Oral Physiological Melanosis Depigmentation: A Case Report Involving Human Papillomavirus

**DOI:** 10.3390/dj12070203

**Published:** 2024-06-30

**Authors:** Leslie Villa-Martínez, Blanca Itzel Mendoza-Espinosa, Luis Fernando Jacinto-Alemán, Adriana Molotla-Fragoso, Claudia Patricia Mejía-Velázquez, Alejandro Alonso-Moctezuma, Carla Monserrat Ramírez-Martínez, David Alonso Trejo-Remigio, Elsa Mónica Toriz-Pichardo

**Affiliations:** 1 Dentistry School, National Autonomous University of Mexico, Mexico City 04510, Mexico; lesly.mcr4@gmail.com; 2 Periodontology Department, Dentistry School, National Autonomous University of Mexico, Mexico City 04510, Mexico; dra.monicatorizp@fo.odonto.unam.mx; 3 Oral Medicine and Pathology Department, Postgraduate and Research Division, Dentistry School, National Autonomous University of Mexico, Mexico City 04510, Mexico; molo.22@fo.odonto.unam.mx (A.M.-F.); dramejiavelazquez@fo.odonto.unam.mx (C.P.M.-V.); c.ramirez@fo.odonto.unam.mx (C.M.R.-M.); icarus268@comunidad.unam.mx (D.A.T.-R.); 4 Oral and Maxillofacial Surgery Department, Postgraduate Division, Dental School, National Autonomous University of Mexico, Mexico City 04510, Mexico; alonsomoctezuma@fo.odonto.unam.mx

**Keywords:** physiological melanosis, gingival hyperpigmentation, gingiva, melanin, HPV infection

## Abstract

Gingiva hyperpigmentation resulting from physiological melanosis causes aesthetic discomfort and is usually perceived as a disease by patients because healthy attached gingiva is typically characterized by coral pink coloring with stippling and scalloped contours. When physiological melanosis compromises the aesthetics of smiling, it may induce insecurity in patients, who usually seek out alternatives for reducing or eliminating hyperpigmentation. We present a case report of a surgical procedure combining gingivectomy with gingivoplasty for the management of physiological melanosis. The surgical procedure was performed on a 40-year-old female patient with bilateral pigmentation in both arches. The results of the histological analysis confirm the diagnoses of melanotic macula, with papillary hyperplasia and cytopathic changes being suggestive of HPV infection, which was verified using an immunohistochemistry analysis based on the detection of a major capsid protein of HPV. Acceptable functional and aesthetic results were obtained for the patient without major discomfort during the postoperative period. In cases when HPV infection is present, long-term follow-up becomes necessary.

## 1. Introduction

The oral mucosa can be divided into three subtypes: masticatory, specialized, and lining mucosa [1,2]. The attached gingiva is a part of the masticatory mucosa that surrounds the teeth and covers the alveolar bone. It extends between the gingival margin and the mucogingival line [3]. The gingiva is one of the four tissues comprising the periodontium. These tissues constitute the support and protection system of the teeth [1,2]. A clinical–anatomical feature characterizing the attached gingiva is its coral pink color, which varies depending on various factors such as keratinization, vascularization, the thickness of the epithelium, and melanin production [1,3].

Melanin is an endogenous brownish-black pigment whose synthesis by melanocytes is stimulated by the pituitary MSH (melanocyte-stimulating hormone). Melanocytes contain melanosomes, the organelles in which melanin pigments are synthesized and stored. TYR and TYRP2 are enzymes that affect the quantity and quality of melanin. There are two types of melanin in mammals: brownish-black eumelanin and reddish-yellow pheomelanin [4]. Their increased synthesis can be observed in diverse skin and mucosa manifestations, such as ephelides, nevi, chloasma that develops in pregnant women, and in physiological melanosis [5,6].

Oral physiological melanosis is a non-pathological condition whose main clinical manifestation is bilateral pigmentation in the attached gingiva without predilection for gender, age, or anatomical site. It is more commonly observed in individuals of African ethnicity or afro-descendant heritage [6]. Although hyperpigmented anatomical structures are normal and functional, their aesthetic appearance can cause problems or discomfort for patients who present them; for this reason, different techniques have been developed to eliminate or reduce gingival hyperpigmentation. Among the reported techniques are cryosurgery, laser therapy, grafts, the use of chemical agents, and gingivectomy with gingivoplasty.

Gingivectomy with gingivoplasty is a surgical technique based on the elimination of the epithelial layer of the attached gingiva and interdental papilla with the goal of achieving healing via secondary intention. This procedure presents favorable results, has short operative and healing times, and has a low cost. However, the main limitations include limited operative visibility due to bleeding as well as operator experience related to the judicious assessment of the depth in gingival epithelium removal [7,8]. A technical advancement that allows for alveolar bone exposition to be avoided involves the use of a rotary instrument, such as a medium-grain diamond bur, to eliminate the remaining pigments in the attached gingiva and interdental papilla. After the surgical procedure, it is necessary to place a surgical dressing and monitor secondary intention healing. The postoperative care of a patient’s indications should include analgesics therapy, performing dental hygiene with an extra soft brush, and 0.2% or 0.12% chlorhexidine rinses [9].

Within the group of viruses that affect the skin and mucosa are human papillomaviruses (HPVs), which mainly cause subclinical infection where there are no visible symptoms. There are more than 220 types of HPV that have been identified, classified as low- (e.g., HPV 1, 2, 6, 8, 11, 34, 40, 42, 43, 44, 61, 69, 71, 72, 81, 83, and 84) and high-risk (e.g., HPV 16, 18, 31, 33, 35, 39, 45, 51, 52, 53, 56, 58, 59, 66, 68, 73, and 82) in terms of cancer development. Clinical benign manifestations related to low-risk virus infections are warts, papillomas, condyloma acuminata, and focal epithelial hyperplasia, while malignant manifestations are cervical, penile, anal, and oropharyngeal cancers. HPV infects the basal keratinocytes of the squamous epithelium through abrasions and microscopic wounds in the skin and mucosa through self-inoculation, vertical transmission from infected mothers to children, or contact through sexual activity. Although high-risk HPV types have been detected in the head and neck regions, more than 80% of these infections are cleared within 6–20 months; the exception is HPV16, which persists for up to 20 months [10,11,12,13].

We present a clinical case involving a surgical procedure of gingivectomy with gingivoplasty combined with diamond burs for oral physiological melanosis hyperpigmentation subclinically infected by HPV.

## 2. Patient Information—Case Presentation

### 2.1. Clinical Findings

**Figure 1:**
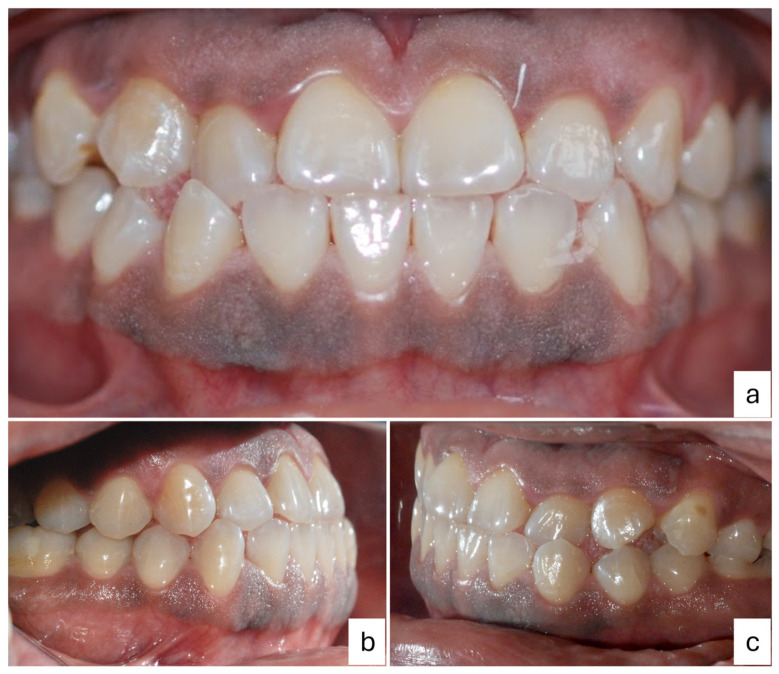
Intraoral photographs of gingival melanosis: (**a**) front view, (**b**) right-side view, and (**c**) left-side view. Brownish-black plaques from premolar to premolar on attached gingiva in both arches.

**Figure 2:**
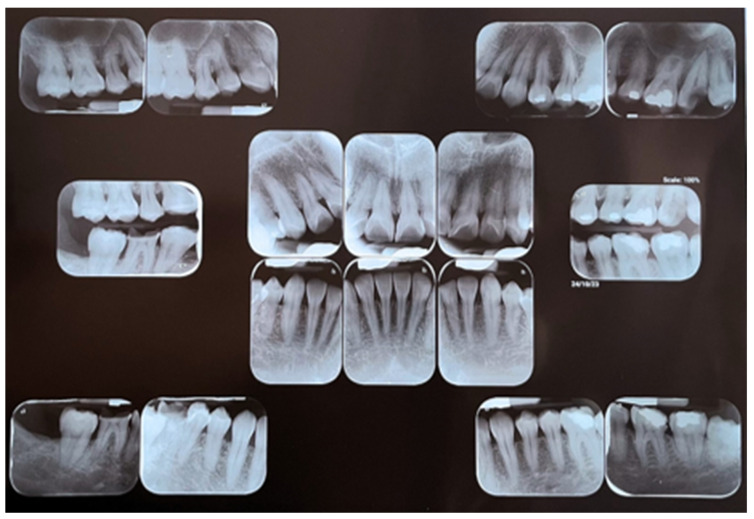
Periapical series where mild loss of bone crest height in lower right molars is observed.

An oral clinical examination of a 40-year-old female patient who attended the Dentistry School of National Autonomous Mexico University revealed moderate dental biofilm accumulation, gingival inflammation, multiple amalgam restorations and temporary fillings, an anterior edge-to-edge bite with occlusal interferences, an altered passive eruption, and bilateral brownish-black plaques on attached gingiva with extension from premolar to premolar in both arches. At palpation, a warty appearance of soft consistency was detected. The patient reported coloration from brownish-black plaques that had been present since they were 8 years old without additional symptoms. A clinical–periodontal diagnosis indicated the localization of periodontitis stage I, grade B, with oral physiological melanosis (Figure 1 and Figure 2). The periodontal treatment plan consisted of Phase I (personal plaque control, brushing technique, calculus removal, and dental polishing) followed by Phase II, which involved a surgical procedure of gingivectomy with gingivoplasty for melanosis depigmentation.

### 2.2. Surgical Procedure

**Figure 3:**
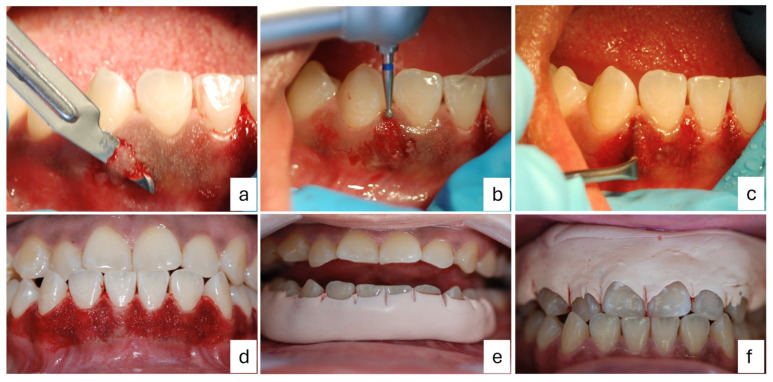
The surgical procedure of gingivectomy with gingivoplasty: (**a**) an external bevel incision for the biopsy, (**b**) the removal of tissue with a ball diamond bur on the interdental papilla, (**c**) gingival remodeling with a Kirkland scalpel, (**d**) exposed connective tissue in the lower arch, (**e**) the placement of surgical dressing in the lower arch, and (**f**) the placement of surgical dressing in the upper arch.

Prior to the local–regional anesthetic infiltration block with 2% lidocaine and a 1:100,000 ratio of epinephrine, the patient rinsed with 0.12% chlorhexidine for 30 s. Tissue removal was performed using a bard parker #3 scalpel and a 15c blade with an internal bevel incision combined with the application of a high-speed # 3 ball diamond bur for the papillary area, and both procedures were performed under constant physiological solution irrigation. The obtained tissue fragments were fixed in 3% formaldehyde solution for a subsequent histopathological analysis. Gingival contour was remodeled using a Kirkland scalpel and washing with physiological solution to place surgical dressing over the entire operated area. The periosteum was left intact since only the epithelium was removed to expose the underlying connective tissue. Postoperative instructions were given to the patient; the patient was prescribed naproxen sodium/paracetamol at a dose of 275 mg/300 mg every 8 h for 3 days and rinsing with 0.12% chlorhexidine after brushing every 12 h for 15 days. After the postoperative period, the surgical dressing was removed without clinical complications, and the surgical procedure for the upper arch was performed following the above protocol (Figure 3).

### 2.3. Clinical and Histopathological Results

**Figure 4:**
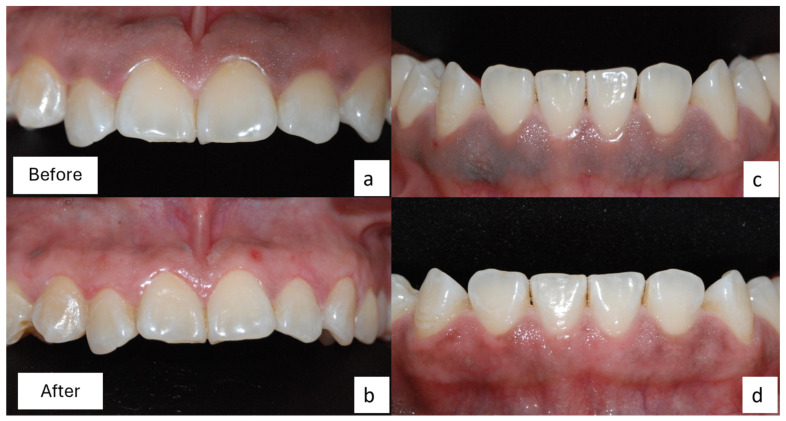
Comparative status of gingival pigmentation. (**a**,**c**) Preoperative features; (**b**,**d**) postoperative features in upper arch taken 8 days after operation and in the lower arch 15 days after operation.

Clinically, a significant decrease in pigmentation was observed in both arches, especially in the upper arch. The above clinical result could be attributed to the removal of less tissue in the lower gingiva due to its thinness. After 3 weeks, the patient reported compliance with the obtained results, with functionality, tolerable thermal perception, and satisfactory hygiene in both arches (Figure 4).

**Figure 5:**
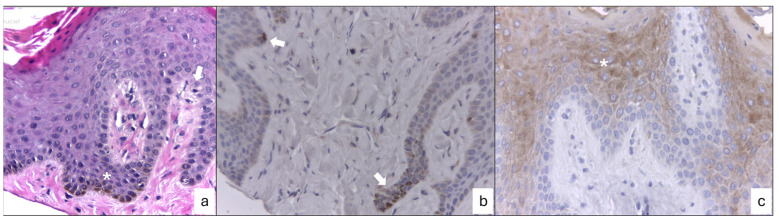
Histopathological and immunohistochemical characteristics. (**a**) Hematoxylin and eosin staining, where brown cells corresponding to melanocytes (*) and coilocytes suggestive of viral infection (arrow) are observed, (**b**) immunoexpression of HMB-45, which confirms the presence of melanocytes (arrows), and (**c**) the immunoexpression of HPV, preferably in the suprabasal strata (*), confirming infection.

Reported in the histopathological analysis are parakeratinized stratified squamous epithelium with an elongated digitiform and anastomosing projections supported by fibrovascular stems as well as the presence of pyknotic nuclei, which were displaced to the periphery by perinuclear vacuole cells or coilocytes in the intermediate and superficial stratus. Pigment deposits corresponding to melanin were observed in the basal stratum, as was melanic incontinence in some areas of underlying dense fibrous connective tissue. The impression diagnoses were of melanotic macula, with papillary hyperplasia and cytopathic changes being suggestive of HPV infection. Peroxidase immunohistochemistry assays were performed for the detection of HMB-45 (sc-59305, Santa Cruz Biotechnology, Paso Robles, CA, USA) in melanocytes and a major capsid protein of human papilloma virus (HPV; BSB-5657, BioSB, Goleta, CA, USA); both antigens were immunopositive, confirming the diagnosis of melanotic macula and HPV infection (Figure 5).

**Figure 6:**
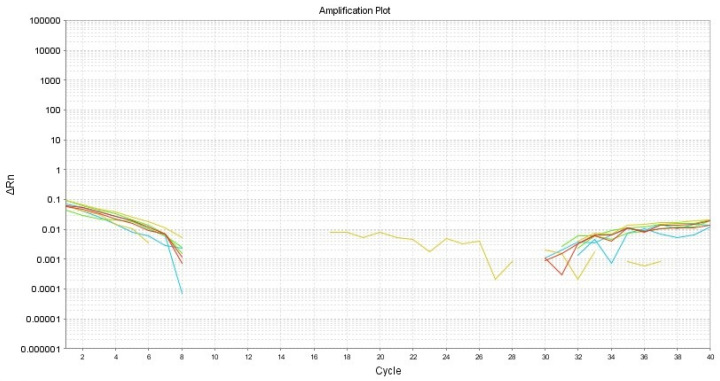
Amplification plot showing non-amplification related to E2, E6, and E7 for HPV16.

Real-time PCR was performed for the identification of HPV16 via the amplification of E2, E6, and E7 and using B2M as an internal control (Table 1). First, DNA extraction from samples for 50 µm slides was performed using ReliaPrep FFPE (Promega, Madison, WI, USA) following the manufacturer’s instructions. To estimate the DNA concentration and purity, a NanoDrop ND-2000 spectrophotometer (Thermo Fisher, Rochester, NY, USA) was employed. qPCR was performed in triplicate using the QuantiNova SYBR Green RT-PCR Kit (Qiagen, Cat. 208052, Hilden, Germany) with a StepOnePlus (Applied Biosystems, Thermo Fisher, Waltham, MA, USA) thermocycler. Relative gene quantification was performed using the 2^−(ΔΔCt)^ method [14]. There was no amplification of the three viral genes in the assay (Figure 6).

## 3. Discussion

Oral physiological melanosis is considered a condition and not a disease, which is why no specific treatment is required. However, due to its aesthetic appearance, affected patients may request therapeutic alternatives. Currently, there are several treatment options, including cryotherapy, laser, chemical agents, and surgery.

Cryotherapy consists of the intentional destruction of soft tissues through the application of extreme cold that leads to freezing. This procedure offers a low possibility of infection, no bleeding, and relatively low pain [15]. A variety of cryogens can be used, including liquid nitrogen (−196 °C), nitrous oxide (−80 °C), solid carbon dioxide or CO_2_ dry ice (−78 °C), and chlorodifluoromethane (−42°), among others [16].

Laser therapy is based on the principle of selective photothermolysis, where laser energy is absorbed by a specific molecule, called a chromophore, generating a controlled response without damaging the surrounding structures [17]. The main chromophores are melanin, hemoglobin, pigmented proteins, and water. When the chromophore absorbs the energy emitted by the laser, there is an increase in the boiling temperature, which generates a microexplosion or ablation, that is, the pigmented cells are destroyed without affecting the non-pigmented ones. This technique must be performed under anesthesia to prevent pain and bleeding, thereby eliminating the need for sutures or periodontal dressing while ensuring a short healing time. Its main disadvantages are the high cost of the laser equipment and the requirement for specialized management to avoid overheating and necrosis. There are several types of lasers, such as carbon dioxide, neodymium-doped yttrium aluminum garnet (Nd:YAG), erbium laser/yttrium aluminum garnet (Er:YAG), and erbium, chromium-doped yttrium, scandium, gallium, and garnet (Er,Cr:YSGG) lasers. Of these, the Nd:YAG laser is most commonly employed in dermatology for pigmented lesion elimination [18,19]. An important consideration is that ablative laser procedures can produce hazardous fumes with toxic, infectious, and carcinogenic effects. Moreover, in the period following the COVID-19 pandemic, this technique should be reconsidered if there is any suspicion of a viral infection [20].

Regarding chemical agents that have been employed to modify melanin pigmentation, ascorbic acid is a reported example. This compound suppresses the formation of dopaquinone, an initiator of melanin synthesis [21]. Other reported compounds are silver nitrate and phenol–alcohol solution; however, both have been linked with secondary adverse effects [16]. Ascorbic acid is used in two methods, namely topical painting of the pigmented region and in-office local injection (oral mesotherapy). Shimada et al. demonstrated that the topical application of ascorbic acid for 4 weeks can reduce gingival pigmentation. Based on the in vitro assays in their study, this effect can be attributed to the inhibition of both tyrosinase activity and melanin formation [21].

Gingivectomy with gingivoplasty is a procedure that offers ease and a low operating cost. To avoid affecting the underlying bone during this procedure, an important feature to consider is the tissue thickness. Our patient presented a thin biotype in the lower arch, with less gingiva removal compared to that of the upper arch. In initial planning within the therapeutic alternatives, the use of some chemical methods was considered; due to economic considerations and patient preferences, however, only the surgical procedure was used.

For all depigmentation approaches, regression or repigmentation could present. The lowest proportion has been reported for cryosurgery (0.32%), followed by electrosurgery (0.74%), laser (1.16%), and gingivoplasty (8.99%), occurring within 3 months to 3 years later [15,18,22,23,24,25]. This heterogeneity in time and the proportion of repigmentation can be attributed to the limitations of the approaches as well as the level or intensity of epithelium and melanin pigmentation in the underlying connective tissue. 

Our histopathological and immunohistochemical analyses confirmed the presence of melanin and melanocytes in the basal stratum, with melanin incontinence in the underlying connective tissue, which is consistent with the diagnosis of melanotic macula. However, an additional finding was observed, namely the presence of coilocytes in the intermediate and superficial epithelium strata, which suggests the presence of human papillomavirus (HPV). The employed antibody reacts with an epitope of a major capsid protein of HPV, which is broadly expressed by the 1, 6, 11, 16, 18, 31,33, 35, 39, 40, 42, 45, 51, 52, 56, 58, 59, 67, and 68 HPV subtypes [26]. Oliveira and Cols reported a low prevalence of HPV infection in the genital and oral mucosa of asymptomatic women, which may be due to the nature of the viral infection being self-limited within 20 months [27]. These viruses can reach the oral mucosa through hand-to-mouth autoinoculation, breastfeeding, and risky sexual practices, and they are possibly related to poor oral health [28]. The detection of infection with HPV types 6 and 11 and melanosis has been reported in the vagina [29] but not in the oral mucosa. As reported by Núñez-Troconis and Cols, physiological melanosis in the vagina is rare, and its presence serves as an alert that a clinical follow-up is needed to detect any malignant changes suggestive of melanoma, especially if HPV is present. In our case, oral physiological melanosis is more common in oral mucosa, and its transformation to melanoma has not been reported [6,16]. However, there are few reports of their malignant transformation in solitary melanotic macules over a 5- to 13-year period [30,31]. Our qPCR results regarding HPV16 detection are negative. Considering the lack of classic clinical characteristics except for immunohistochemical positivity, it is possible that it is a low-risk HPV, but this should be verified through additional tests. 

It is important to be cautious with respect to clinical interrogation when obtaining adequate data and informing patients about the need for constant clinical follow-ups not only to monitor pigmentation changes, but also to monitor papillomatous modifications in the gingival surface, as a histopathological analysis is needed to detect any malignant alterations of cellular pleomorphism, atypical mitosis, and melanocytic hyperplasia.

## 4. Conclusions

Gingivectomy with gingivoplasty is an excellent alternative procedure for patients who are aesthetically affected by physiological melanosis. It does not generate greater postoperative discomfort and produces acceptable results for patients. However, long-term follow-up is required to monitor possible recurrence.

Although our patient was clinically asymptomatic regarding HPV infection, which may spontaneously resolve within 2 years, the histopathological findings indicate its presence, so clinical follow-ups for that period of time would assist in finalizing a comprehensive treatment strategy for the patient’s benefit.

## Figures and Tables

**Table 1:** PCR primer set.

Gene	Primer Sequence	GenBank Accession
B2M-Forward	AATGCTTGGCTGTGATAC	NG_012920.2
B2M-Reverse	CTATGGCGGAAGATAACTG	
E2-Forward	AGGACGGATTAACTGTAA	OP712097.1
E2-Reverse	GTTGCCATTCACTATCATA	
E6-Forward	ATTAGAACAGCAATACAACAA	
E6-Reverse	GCAACAAGACATACATCG	
E7-Forward	ACAGAGCCCATTACAATA	
E7-Reverse	CATTAACAGGTCTTCCAA	

## Data Availability

The data presented in this study are available upon request from the corresponding author.
